# Psychological, physical, and sleep comorbidities and functional impairment in irritable bowel syndrome: Results from a national survey of U.S. adults

**DOI:** 10.1371/journal.pone.0245323

**Published:** 2021-01-14

**Authors:** Madhusudan Grover, Bhanu Prakash Kolla, Rahul Pamarthy, Meghna P. Mansukhani, Margaret Breen-Lyles, Jian-Ping He, Kathleen R. Merikangas

**Affiliations:** 1 Division of Gastroenterology and Hepatology, Mayo Clinic, Rochester, MN, United States of America; 2 Department of Psychiatry & Center for Sleep Medicine, Mayo Clinic, Rochester, MN, United States of America; 3 Department of Family Medicine & Center for Sleep Medicine, Mayo Clinic, Rochester, MN, United States of America; 4 Genetic epidemiology Research Branch, National Institute of Mental Health, Bethesda, MD, United States of America; IRCCS Istituto Delle Scienze Neurologiche di Bologna, ITALY

## Abstract

**Background/Aims:**

Patients with irritable bowel syndrome (IBS) in referral practice commonly report mental disorders and functional impairment. Our aim was to determine the prevalence of mental, physical and sleep-related comorbidities in a nationally representative sample of IBS patients and their impact on functional impairment.

**Methods:**

IBS was defined by modified Rome Criteria based on responses to the chronic conditions section of the National Comorbidity Survey-Replication. Associations between IBS and mental, physical and sleep disorders and 30-day functional impairment were examined using logistic regression models.

**Results:**

Of 5,650 eligible responders, 186 met criteria for IBS {weighted prevalence 2.5% (SE = 0.3)}. Age >60 years was associated with decreased odds (OR = 0.3; 95% CI:.1-.6); low family income (OR = 2.4; 95% CI:1.2–4.9) and unemployed status (OR = 2.3; 95% CI:1.2–4.2) were associated with increased odds of IBS. IBS was significantly associated with anxiety, behavior, mood disorders (ORs 1.8–2.4), but not eating or substance use disorders. Among physical conditions, IBS was associated with increased odds of headache, chronic pain, diabetes mellitus and both insomnia and hypersomnolence related symptoms (ORs 1.9–4.0). While the association between IBS and patients’ role impairment persisted after adjusting for mental disorders (OR = 2.4, 95% CI 1.5–3.7), associations with impairment in self-care, cognition, and social interaction in unadjusted models (ORs 2.5–4.2) were no longer significant after adjustment for mental disorders.

**Conclusion:**

IBS is associated with socioeconomic disadvantage, comorbidity with mood, anxiety and sleep disorders, and role impairment. Other aspects of functional impairment appear to be moderated by presence of comorbid mental disorders.

## Introduction

Irritable bowel syndrome (IBS) affects up to 15% of U.S. adults and results in significant impairment in quality of life, loss of work productivity and increased healthcare utilization [1]. Patients with IBS have a higher prevalence of physical comorbidity including migraine headaches, fibromyalgia, temporomandibular joint dysfunction, chronic pelvic pain and interstitial cystitis [2, 3]. A diagnosis of IBS has been associated with lower socioeconomic status in some [4] but not all studies [5].

Psychiatric symptoms and disorders are an independent risk factor for the development of IBS [6] and can exacerbate or perpetuate symptoms [7]. Anxiety disorders can precipitate or worsen symptoms through associated heightened autonomic arousal (in response to stress) or through changes in gastrointestinal (GI) sensitivity and motor function [7]. Somatization disorders are also associated with gastric [8] and rectal hypersensitivity [9]. Additionally, psychosocial factors can impact the ability to cope with functional GI symptoms and have been linked to worse outcomes and quality of life [10].

Poor sleep quality, prolonged sleep latency, frequent nighttime awakenings, and daytime dysfunction are also commonly associated with IBS, similar to that seen in patients with inactive inflammatory bowel disease [11]. IBS patients report greater use of hypnotic medications [11]. Several studies have shown impairment in both subjective and objective sleep measures in IBS patients. Both increased [12, 13] and decreased [14] rapid eye movement (REM) sleep have been reported in IBS. Additionally, greater sleep fragmentation correlated with daytime sleepiness and impaired quality of life in IBS patients [15]. However, the prevalence of insomnia and hypersomnolence related symptoms, defined using established diagnostic criteria in a community sample of individuals with IBS, have not been documented.

Patterns of comorbidity between psychiatric and medical conditions have not been documented in the general population of the U.S. Additionally, there is limited knowledge regarding functional impairment or patterns of comorbidity among those with IBS in the general population. Therefore, the aim of this study is to determine the prevalence of comorbidity with mental, physical, and sleep disorders, and associated functional impairment in a large representative sample of community dwelling U.S. adults with IBS.

## Materials and methods

### Sample

This study was Institutional Review Board (IRB) exempt as data from the National Comorbidities Survey- Replication were analyzed anonymously. The National Comorbidity Survey-Replication (NCS-R), a national survey of individuals ≥18 years of age was designed to represent non-institutionalized English speaking adults living in households or campus group housing in the coterminous U.S. (i.e. excluding Alaska and Hawaii) [16]. The NCS-R was a face-to-face interview conducted between February 2001 and December 2003 and it sampled 9,282 participants with a response rate of 70.9%. The NCS was approved by the Institutional Review Board of Harvard Medicine School (IRB E093098-2) on September 30, 1998. The study protocol conforms to the ethical guidelines of the 1975 Declaration of Helsinki as reflected in *a priori* approval by the institution’s human research committee. Additional sampling details are published elsewhere [17] and provided in **S1 File**.

### Measures

#### Irritable bowel syndrome

The chronic conditions section of NCS-R part II included three primary questions on bowel function (“Have you ever had a period lasting 12 months or longer when at least one week each month you had frequent pain or discomfort in your stomach or lower abdomen that was relieved when you had a bowel movement?”, “Did you have either frequent diarrhea or frequent constipation during that period?”, and “Did you have a change in the frequency of your bowel movements during this period?”). Subjects with a “yes” response for the pain question and a “yes” on either of the subsequent two questions were considered to have “IBS” with modified Rome Criteria. These questions are similar to but require presence of “frequent” symptoms persisting for a longer duration (≥12 months) than the Rome criteria [18, 19] and hence participants were classified as having IBS with modified Rome Criteria.

#### Demographic and social variables

Information regarding socio-demographic variables including age (18–29, 30–44, 45–59 and >60 years), sex, race/ethnicity (non-Hispanic black, non-Hispanic white, Hispanic, other), employment status (working, student, homemaker, retired, other) and family income (low, <1.5 times of federal poverty line; low-average, 1.5–3.0 times of poverty line; high-average, >3.0–6.0 times of poverty line; and high, > 6.0 times of poverty line) were collected.

#### Mental disorders

Core DSM-IV anxiety disorders (agoraphobia, generalized anxiety disorder, panic disorder, social phobia, specific phobia, posttraumatic stress disorder, separation anxiety disorder), behavior disorders (attention deficit hyperactivity disorder, conduct disorder, oppositional defiant disorder), eating disorders (anorexia, bulimia, binge eating), mood (major depressive disorder, dysthymia, bipolar I or II), and substance use disorders (alcohol and drug abuse/dependence) were identified based on the responses to the Part I CIDI interview. DSM-IV diagnoses ascertained by CIDI interview have been shown to be concordant with those established by independent clinical assessment [20].

#### Physical conditions

Presence of chronic physical conditions was determined from the part II interview based on self-report. Chronic physical conditions were further classified as headaches, chronic pain (which included chronic neck/back pain), stroke, heart attack, heart disease, hypertension, chronic lung disease, diabetes mellitus, epilepsy and cancer.

#### Sleep disorders

Subjects who endorsed sleepiness as determined by their response to the question “Did you have a period lasting two weeks or longer in the past 12 months when you had problems feeling sleepy during the day?” were then queried about “their tendency to fall asleep in permissive situations (while watching TV, listening to the radio, reading, within 10 minutes of sitting still, during conversations or while visiting friends)”; “if they felt that they had not slept enough despite spending enough time in bed”; or “if they had difficulty getting up in the morning.” Those who endorsed feeling sleepy during the day and had one additional sub-symptom were considered to have “hypersomnolence-related symptoms”. A response to “Do you have difficulty with getting to sleep, staying asleep or waking too early?” was considered for “insomnia-related symptoms” [21]. Subjects who endorsed having these symptoms “often” or “sometimes” were considered to have a positive response. Information on sleep duration was not collected in NCS-R.

#### Functional impairment

The WHO Disability Assessment Schedule 2.0 (WHODAS-II) was administered to all participants in the Part II sample, asking about disability attributable to health, emotional or mental health problems. The WHODAS-II assesses functional impairments in several domains in the past 30 days: self-care (e.g., bathing, dressing), mobility (e.g., standing, walking), cognition (e.g., concentrating, remembering), social functioning (e.g., conversing, maintaining emotional control while around others), and role impairment (e.g., quality and quantity of normal activities at home or work). All five individual WHODAS-II disability domain scores were transformed to a theoretical range of 0 (no impairment) to 100 (complete inability to function) and were aggregated to calculate a global score.

### Statistical analysis

Cross tabulations were used to calculate the distribution of IBS prevalence by demographic and social variables. Multivariate logistic regression models were used to examine the associations between IBS status and dichotomous variables including DSM-IV mental disorders, physical conditions, sleep disorders and functional impairment (WHODAS-II scores), in which IBS status was treated as dichotomous exposure of interest. Separate regression models were run progressively adjusting for 1) demographic characteristics, and 2) other classes of DSM-IV mental disorders. Logistic regression coefficients and their standard errors were exponentiated and presented as odds ratios (OR) with 95% confidence intervals. All statistical analyses were completed with the SAS version 9.4 and SUDAAN version 11 using the Taylor series linearization method to take into account the complex survey design (see **S1 File**). All statistical significance was based on two-sided tests evaluated at the .05 level of significance.

## Results

A total of 5,692 individuals constituted the overall NCS-R Part II sample. Of these, 42 respondents endorsed a history of ulcerative colitis or Crohn’s disease and were removed from further analyses. Of the 5,650 eligible responders, 186 met modified Rome Criteria for IBS as defined previously. This provided a weighted point prevalence of 2.5% {standard error (SE) = 0.30}.

### Demographic and social variables

**Table 1** details the demographic and social characteristics of subjects with IBS. Prevalence differences in females and males did not reach statistical significance in this cohort. IBS was less prevalent in individuals >60 years of age (0.9%, SE = 0.2) as compared to the reference (18–29 years old) population (2.9%, SE = 0.5). The prevalence of IBS did not differ based on ethnicity. Subjects with a low family income (<1.5 times of poverty line) had a higher prevalence of IBS compared to those with high family income (>6 times of poverty line) (OR = 2.39; 95% CI, 1.17–4.90). Compared to those who were working, subjects who were unemployed had a greater prevalence of IBS (OR = 2.28; 95% CI, 1.24–4.19) and subjects who were retired/homemakers/students had a lower prevalence (OR = 0.42; 95% CI, 0.25–0.72).

**Table 1:** Demographic and social characteristics of Irritable Bowel Syndrome in NCS-R Part II survey.

Characteristics			IBS	
		N	N of cases, % (SE)	OR (95% CI)
Total		5,650	186, 2.5 (0.3)	-
Sex	Male	2,368	79, 2.2 (0.3)	Reference
	Female	3,282	107, 2.8 (0.4)	1.27 (0.90–1.79)
Age Group	18–29 yrs	1,365	53, 2.9 (0.5)	Reference
	30–44 yrs	1,815	76, 3.6 (0.6)	1.22 (0.78–1.93)
	45–59 yrs	1,507	43, 2.1 (0.4)	0.71 (0.41–1.23)
	60+ yrs	963	14, 0.9 (0.2)	**0.30 (0.15–0.61)**
Race/Ethnicity	Hispanic	525	15, 1.7 (0.6)	0.68 (0.35–1.34)
** **	Non-Hispanic Black	713	22, 3.6 (1.3)	1.48 (0.71–3.09)
	Other	266	12, 2.0 (0.8)	0.83 (0.37–1.86)
	Non-Hispanic White	4,146	137, 2.4 (0.2)	Reference
Family Income ^a^	Low	1,169	48, 3.4 (0.7)	**2.39 (1.17–4.90)**
	Low average	1,257	49, 2.3 (0.5)	1.61 (0.72–3.59)
	High average	1,871	65, 2.7 (0.3)	1.84 (0.99–3.42)
	High	1,353	24, 1.5 (0.4)	Reference
Employment Status	Working	3,896	127, 2.5 (0.3)	Reference
	Retired/homemaker/student	1,150	22, 1.1 (0.2)	**0.42 (0.25–0.72)**
	Unemployed	604	37, 5.6 (1.5)	**2.28 (1.24–4.19)**

### Mental disorders

The prevalence of DSM-IV anxiety, behavior and mood disorders was higher in those with IBS (**Table 2**). A total of 37% of those with IBS fulfilled criteria for anxiety disorders and 27% for mood disorders. However, prevalence of eating disorders (1–2.5%) and substance use disorders (13–20%) was similar among those with and without IBS. The increased odds (anxiety OR 3.16; 95% CI, 2.18–4.58; behavior OR 4.4; 95% CI, 2.29–8.44; and mood disorders OR 4.22; 95% CI, 2.8–6.37) persisted after adjusting for demographic variables (sex, age, family income, and employment status) and the presence of other DSM-IV mental disorders.

**Table 2:** Prevalence, unadjusted and adjusted risk of DSM-IV mental disorders in the adult IBS cohort in the NCS-R Part II survey.

DSM-IV Mental Disorder (past 12 months)	N with mental disorder	Irritable Bowel Syndrome		Odds Ratio (95% CI)*		
		No	Yes	Unadjusted^a^	Adjusted for demographics^b^	Adjusted for comorbidity^c^
		% (SE)	% (SE)	(Reference: IBS = No)		
Anxiety	1,466	15.7 (0.6)	37.1 (4.2)	**3.16 (2.18–4.58)**	**2.53 (1.66–3.88)**	**1.78 (1.13–2.80)**
Behavior	238	2.8 (0.2)	11.2 (2.8)	**4.40 (2.29–8.44)**	**3.32 (1.65–6.67)**	**2.22 (1.25–3.93)**
Eating	87	1.0 (0.1)	2.5 (1.1)	2.41 (0.90–6.45)	2.03 (0.72–5.72)	1.24 (0.43–3.56)
Mood	817	8.1 (0.3)	27.2 (3.9)	**4.22 (2.80–6.37)**	**3.27 (2.09–5.12)**	**2.38 (1.53–3.71)**
Substance Use	960	13.2 (0.7)	19.9 (3.6)	1.63 (0.98–2.71)	1.31 (0.77–2.24)	0.92 (0.51–1.67)
**Any**	2,387	28.7 (1.0)	54.0 (4.4)	**2.91 (1.98–4.29)**	**2.34 (1.55–3.53)**	**2.34 (1.55–3.53)**

a = unadjusted

b = adjusted for demographic characteristics (sex, age, family income, employment status)

c = additionally adjusted for any DSM-IV mental disorder(s).

### Physical conditions

Subjects with IBS reported higher prevalence of headaches (OR 3.23; 95% CI, 2.21–4.72), chronic pain (OR 3.54; 95% CI, 2.29–5.45) and diabetes mellitus (OR 2.45; 95% CI, 1.27–4.75) (**Table 3**); 48% of those with IBS reported headaches, 63% chronic pain and 16% diabetes mellitus. The associations between IBS and medical conditions persisted even after adjustment for demographic characteristics and for having a DSM-IV mental disorder. The prevalence of stroke, heart attack, heart disease, chronic lung disease, epilepsy and cancer were similar among individuals with and without IBS.

**Table 3:** Prevalence, unadjusted and adjusted risk of chronic medical conditions in the adult IBS cohort in the NCS-R Part II survey.

Physical conditions	N with physical condition	Irritable Bowel Syndrome		Odds Ratio (95% CI)*		
		No	Yes	Unadjusted^a^	Adjusted for demographics^b^	Adjusted for comorbidity^c^
		% (SE)	% (SE)	(Reference: IBS = No)		
Headache	1645	22.0 (0.8)	47.6 (4.7)	**3.23 (2.21–4.72)**	**2.78 (1.82–4.26)**	**2.18 (1.41–3.38)**
Chronic back or neck pain	1889	28.5 (0.8)	54.9 (4.5)	**3.05 (2.08–4.48)**	**3.27 (2.22–4.83)**	**2.74 (1.88–4.00)**
Stroke	149	2.7 (0.3)	2.0 (0.8)	0.75 (0.31–1.82)	0.74 (0.30–1.81)	0.62 (0.25–1.55)
Heart attack	185	3.7 (0.4)	3.2 (1.5)	0.86 (0.33–2.21)	1.11 (0.43–2.87)	0.89 (0.31–2.55)
Heart disease	312	5.0 (0.4)	4.5 (1.8)	0.90 (0.41–2.01)	1.28 (0.60–2.74)	1.01 (0.46–2.24)
Hypertension	1372	23.9 (0.6)	31.6 (5.2)	1.47 (0.91–2.38)	**2.14 (1.34–3.42)**	**1.89 (1.15–3.11)**
Chronic lung dx	144	2.1 (0.3)	5.1 (2.9)	2.51 (0.68–9.23)	2.64 (0.86–8.12)	2.11 (0.73–6.10)
Diabetes	412	7.0 (0.4)	15.6 (4.1)	**2.45 (1.27–4.75)**	**3.10 (1.55–6.21)**	**2.94 (1.47–5.86)**
Epilepsy	134	1.7 (0.2)	5.1 (3.4)	3.04 (0.77 11.96)	2.18 (0.68–6.99)	2.02 (0.53–7.69)
Cancer	375	6.6 (0.5)	3.6 (1.5)	0.53 (0.23–1.18)	0.87 (0.37–2.08)	0.74 (0.30–1.83)
**Any**	3704	59.7 (0.7)	79.5 (4.2)	**2.62 (1.56–4.40)**	**3.30 (2.04–5.32)**	**2.71 (1.67–4.38)**

a = unadjusted

b = adjusted for demographic characteristics (sex, age, family income, employment status)

c = additionally adjusted for any DSM-IV mental disorder(s).

### Sleep disorders

The odds of having insomnia-related symptoms along with hypersomnolence-related symptoms (OR 5.63; 95% CI, 3.48–9.11), hypersomnolence-related symptoms alone (OR 4.93; 95% CI, 2.7–9.01) or insomnia-related symptoms alone (OR 3.20; 95% CI, 1.69–6.03) were higher in those with IBS than in controls (**Table 4**). Seventy three percent of subjects with IBS reported having a sleep disorder compared to 37% of the controls. The increased odds of having a sleep disorder persisted even after adjusting for demographic characteristics and for the presence of any DSM-IV mental disorder.

**Table 4:** Prevalence, unadjusted and adjusted risk of symptom-based sleep disturbances in the adult IBS cohort in the NCS-R Part II survey.

Sleep symptoms	N with physical condition	Irritable Bowel Syndrome		Odds Ratio (95% CI)*		
		No	Yes	Unadjusted^a^	Adjusted for demographics^b^	Adjusted for comorbidity^c^
		% (SE)	% (SE)	(Reference: IBS = No)		
Insomnia + hypersomnolence related symptoms	1096	13.9 (0.6)	33.9 (3.9)	**5.63 (3.48–9.11)**	**4.95 (3.07–8.01)**	**3.65 (2.16–6.17)**
Hypersomnolence related symptoms	630	8.7 (0.6)	18.6 (3.5)	**4.93 (2.70–9.01)**	**4.62 (2.50–8.53)**	**4.00 (2.15–7.44)**
Insomnia related symptoms	920	14.6 (0.6)	20.2 (4.9)	**3.20 (1.69–6.03)**	**3.09 (1.78–5.36)**	**2.67 (1.52–4.69)**
Neither	3004	62.9 (1.1)	27.3 (4.2)	**Reference**	**Reference**	**Reference**

a = unadjusted

b = adjusted for demographic characteristics (sex, age, family income, employment status)

c = additionally adjusted for any DSM-IV mental disorder(s) in the last 12 months.

### Functional impairment

The responses in functional impairment domains showed that people with IBS had greater impairments in self-care (OR 2.46; 95% CI, 1.38–4.36), cognition (OR 2.59; 95% CI, 1.51–4.44), social interaction (OR 3.79; 95% CI, 2.37–6.04) and role impairment (OR 4.16; 95% CI, 2.17–7.97). Those with IBS did not have greater impairment in their mobility. Impairment in self-care and cognition were no longer significant after adjustments for demographic variables. Social impairment persisted after adjustment for demographic variables but was no longer significant after adjustment for DSM-IV mental disorders. However, the association between IBS and role-impairment persisted in all adjusted models (**Table 5**).

**Table 5:** Domain and overall scores for functional impairment (WHODAS-II) in the adult IBS cohort in NCS-R Part II survey.

Disability^+^	N (≥95th percentile)	Irritable Bowel Syndrome		Odds Ratio (95% CI)*		
		No	Yes	Unadjusted^a^	Adjusted for demographics^b^	Adjusted for comorbidity^c^
		% (SE)	% (SE)	(Reference: IBS = No)		
Self-Care	276	3.9 (0.3)	9.1 (2.1)	**2.46 (1.38–4.36)**	1.88 (0.91–3.87)	1.56 (0.80–3.04)
Mobility	297	4.6 (0.3)	10.3 (4.1)	2.40 (0.97–5.96)	1.94 (0.82–4.57)	1.68 (0.67–4.26)
Cognition	280	3.1 (0.2)	7.7 (1.8)	**2.59 (1.51–4.44)**	1.88 (0.99–3.58)	0.91 (0.51–1.63)
Social interaction	288	3.1 (0.3)	10.9 (2.0)	**3.79 (2.37–6.04)**	**2.91 (1.60–5.29)**	1.57 (0.92–2.68)
Role impairment	351	4.8 (0.4)	17.4 (4.5)	**4.16 (2.17–7.97)**	**3.12 (1.82–5.35)**	**2.39 (1.23–4.64)**
**Global score**	280	3.8 (0.4)	10.1 (3.6)	**2.82 (1.22–6.50)**	1.78 (0.79–3.98)	1.28 (0.46–3.56)

+ World Health Organization Disability Scale-II: 95% percentiles cutoffs for WHODAS-II scores = 27.8 for global, 1.39 for self-care, 50 for mobility, 9.38 for cognition, 3.3 for social interaction, and 100 for role impairment.

^a^unadjusted

^b^adjusted for demographic characteristics (sex, age, family income, employment status)

^c^additionally adjusted for DSM-IV mental disorder(s).

### Health care utilization

Direct measures of health care utilization were not available in the NCS-R. However, participants were asked about services received in the past year. These were categorized as mental health, general medical, human services, complementary and alternative medicine services. Overall, patients with IBS utilized significantly greater services than those without IBS (OR 3.53; 95% CI, 2.28–5.47); however, the use of complementary and alternative medicine services was no different than those without IBS (OR: 0.57; 95% CI, 0.30–1.09). After adjusting for comorbidities, this remained higher than non-IBS participants (OR 2.10; 95% CI, 1.37–3.21); however, the use of complementary and alternative medicine services was no different than those without IBS (OR: 0.57; 95% CI, 0.30–1.09). (**Table 6**).

**Table 6:** Health-care services obtained in the past year.

Services in the past year	N of respondents received service in the past year	Irritable Bowel Syndrome		OR (95% CI)		
		No	Yes	Unadjusted^a^	Adjusted for demographics^b^	Adjusted for comorbidity^c^
		% (SE)	% (SE)	(Reference: IBS = No)		
Mental Health Specialty	731	8.5 (0.5)	17.6 (2.8)	**2.29 (1.54–3.40)**	**1.79 (1.18–2.71)**	1.06 (0.65–1.75)
General Medical	766	8.8 (0.3)	26.5 (5.0)	**3.72 (2.18–6.33)**	**3.33 (2.01–5.51)**	**2.24 (1.29–3.89)**
Human Services	266	3.2 (0.2)	9.8 (2.8)	**3.24 (1.74–6.02)**	**2.53 (1.35–4.74)**	1.75 (0.93–3.31)
Complementary & Alternative Medicine	244	2.8 (0.2)	3.3 (0.8)	1.18 (0.68–2.04)	0.94 (0.54–1.61)	0.57 (0.30–1.09)
**Any service**	1467	17.3 (0.7)	42.5 (5.2)	**3.53 (2.28–5.47)**	**3.00 (1.95–4.62)**	**2.10 (1.37–3.21)**

Mental Health Specialty = Outpatient, inpatient and emergency room services; General Medical = Family doctors; Human services = Home counseling, spiritual advisors, hotline; Complementary & Alternative Medicine = Self-help groups, healers, chiropractors.

^a^unadjusted

^b^adjusted for demographic characteristics (sex, age, family income, employment status)

^c^additionally adjusted for DSM-IV mental disorder(s).

## Discussion

This study examined the mental and physical comorbidity and functional impairment associated with IBS in a large population-based sample in the United States based on a face-to-face household interview. The prevalence of IBS was higher in younger individuals, those who were unemployed and had lower family income. People with IBS were more likely to have anxiety, behavior and mood disorders, but not eating or substance use disorders. Physical conditions that were associated with IBS included headaches, chronic pain and diabetes mellitus. Sleep disturbances were present in a substantial proportion of subjects with IBS, with a majority reporting both hypersomnolence-related and insomnia-related symptoms. Finally, people with IBS also had greater functional impairment than controls, particularly role impairment that was not attributable to comorbid mental disorders.

Although IBS prevalence as low as 1.1% has previously been described [22], the prevalence of 2.5% in this study is lower than recent estimates from North America (~pooled prevalence 5.3%) [23]. The same systematic review found variations in IBS prevalence by region, definition used, and assessment type. This lower than expected prevalence is likely due to differences in the questions used in the NCS-R survey and the conventional Rome questionnaire. For example, NCS-R chronic conditions section inquired about “frequent” pain/discomfort period lasting 12 month or longer. This is different than Rome questionnaire where pain 2-3/month (Rome III) or once/week (Rome IV) is sufficient to reach the threshold. Similarly, “sometimes (≥25%)” is the required threshold for bowel symptoms in Rome compared to “frequent” in the NCS-R. Finally, Rome criteria ask for pain in last 3 months as compared to the “12-month period” in the NCS-R, making NCS-R threshold harder to reach. Additionally, this was a broader questionnaire with only a selected set of gastrointestinal questions. It is possible that questionnaires dedicated specifically for assessment of IBS may provide a higher prevalence due to response bias. Similar to prior studies in the U.S. [4], those with IBS were more likely to be unemployed and had lower incomes than those without IBS. The U.S. Householder survey also found lower household income to be associated with greater reporting of functional gastrointestinal disorders. However, employment status was not found to be significantly associated with a diagnosis of IBS in that study [24]. However, socioeconomic status was not associated with IBS in other epidemiologic studies conducted worldwide [5]. It is unclear if sociocultural factors variably influence disease presentation or if there is geographic variability in the socio-economic consequences of having a chronic disease such as IBS.

Psychological distress is an independent risk factor for development of IBS and can exacerbate or perpetuate symptoms [7]. Anxiety is present in 30–50% of patients with functional GI disorders [7]. Somatization or multiple somatic symptom disorder is present in about two-thirds of patients with IBS and other functional GI disorders and is associated with other concurrent psychiatric diagnoses [3]. The presence of these comorbid conditions may also increase the severity of IBS symptoms [25]. Our results are aligned with the existing literature and suggest that about a third of people with IBS have anxiety or mood disorders. IBS was not associated with eating disorders, suggesting that these conditions do not commonly co-occur. Finally, although up to 20% of IBS subjects reported substance use disorders compared to 13% of the remainder, these proportions were not statistically different.

Our findings also confirm previous studies of medical comorbidity in IBS which have shown that individuals with IBS have 1.4–1.5 times higher odds of migraine, fibromyalgia and depression [26]. A national insurance database study of 125,000 IBS patients found that the prevalence of migraine in patients with IBS was 60 per 1000 versus 22 per 1000 in the control population [26]. Other smaller studies have shown that a quarter to half of IBS patients report migraine [27, 28]. A meta-analysis showed that IBS patients have coexisting headache with an estimated OR of 2.7 (CI 2.3–3.1) [29]. A recent study showed that migraine is also a risk factor for the development of IBS, even in the absence of comorbid anxiety or depression [30]. Other pain disorders are also common in patients with IBS. A health insurance database study from Taiwan showed that after adjustment for age, sex and comorbidity, fibromyalgia was associated with a 1.5-fold increased risk for IBS [31]. Similar results for fibromyalgia have been demonstrated in studies conducted in the U.S. [26] Our study also showed a higher prevalence of migraine and chronic pain in people with IBS.

Sleep disruptions appear to be common in patients with IBS. While one study showed that self-reported sleep disturbances were more common in men with IBS [32], another showed an equal prevalence in men and women [33]. A strong positive correlation was observed between severity of sleep disturbances and IBS symptoms in two studies [11, 32]. Objective measures of sleep demonstrate that IBS patients have prolonged sleep onset latency, increased percentage of non-REM stage 2 sleep, increased latency to first REM sleep, increased arousals and awakenings and wake time after sleep onset, decreased total sleep time and sleep efficiency (percentage of time in bed that is spent asleep). One small study simultaneously assessing small bowel motility and polysomnography showed that IBS patients had similar motility patterns as controls during sleep, but half of the patients with IBS experienced episodes of sleep apnea [13]. Another study found that sleep fragmentation is significantly greater in IBS patients and correlated with daytime sleepiness and impairment in overall quality of life [15]. We found that both insomnia-related and hypersomnolence-related symptoms are common in subjects with IBS, with up to 70% reporting symptoms consistent with at least some degree of sleep disturbance. The increased risk for sleep disturbances persisted after adjusting for mental disorders. These findings suggest that sleep disorders are extremely common in IBS and may potentially be targets for intervention in order to improve overall outcomes.

Studies of clinical samples have shown that IBS is associated with poor general health, higher levels of social and general role impairment scores [34], impaired physical and mental functioning [35], and a high level of avoidance in a range of daily activities, particularly in the presence of abdominal pain [36]. Our results confirm these findings of increased functional impairment associated with IBS in a general community sample in the U.S. However, after adjustment for demographic characteristics and mental disorders, only role impairment was significantly associated with having IBS. This suggests that a significant portion of the functional impairment in subjects with IBS is likely driven by psychiatric comorbidity. Additionally, limitations in life activities observed in IBS patients may be dependent on severity of pain or bowel dysfunction. Thus, addressing psychiatric comorbidity in patients with IBS may reduce the level of functional impairment. For example, in selected patients, atypical antipsychotics such as quetiapine may have beneficial effects on IBS symptoms as well as sleep architecture [37, 38]. Our study also found greater utilization of medical and mental health care services by patients with IBS. Interestingly, the use of complementary and alternative medicine services was not higher than in the non-IBS population. Similar observations were made in the U.S. Householder survey where patients with functional GI disorders had higher rates of physician visits both for GI and non-GI illnesses [24].

This study has significant strengths. We believe that this is the first assessment of the prevalence of IBS and associated mental and physical comorbidity based on a face-to-face interview of a nationally representative community dwelling sample in the United States. The diagnosis of mental disorders was based on an exhaustive in-person interview. The assessments for functional impairment utilized a comprehensive and well validated scale. We adjusted for multiple covariates in our analyses of the associations between IBS and mental and physical disorders and impairment, thereby distinguishing whether these associations were associated with IBS or attributable to demographic or comorbid mental conditions.

Our study must also be viewed in light of some limitations. The gastrointestinal questions in NCS-R survey did not use validated Rome questionnaire or allow determination of subtypes of IBS (diarrhea or constipation predominant). Although patients with inflammatory bowel disease were excluded, other diseases such as celiac disease or microscopic colitis that can cause IBS like symptoms were not specifically delineated in NCS-R. However, frequent pain seen in IBS is less common in those disorders. Objective measurement of sleep parameters was not possible in this survey-based assessment. Additionally, presence of medical comorbidities is established based on self-report. Lack of objective validation may result in inaccurate estimates; however, these factors should be equally distributed among IBS patients and controls. Other limitations pertaining to a survey-based study including responder and recall biases are relevant to this study. While the diagnosis of IBS was based on responses to the chronic conditions section applied to the subject’s entire lifetime, functional impairment was assessed only over the last 30 days. Finally, this study provides evidence of a cross sectional association so directional or causative inferences cannot be drawn. Regardless, this report represents a large population-based study of community dwelling adults in the U.S. who were interviewed in an unbiased manner from the standpoint of a diagnosis of IBS.

## Conclusions

In conclusion, we show that IBS is associated with both socioeconomic disadvantage and several physical and psychiatric conditions. Some but not all aspects of functional impairment in IBS appear to be driven by comorbid mental conditions. From a clinical perspective, addressing sleep-related symptoms and psychiatric disorders may improve the morbidity and functional impairment associated with IBS. Additionally, since not all aspects of functional impairment in IBS are dependent on comorbid mental disorders, targeting pain and motility associated symptoms of IBS is critical to fully optimize the care and restore functioning. Further validation studies in prospective cohorts are needed to delineate factors that drive functional impairment in IBS.

## Supporting information

S1 File (DOCX)
