# Supplementary material for: Psychological, physical, and sleep comorbidities and functional impairment in irritable bowel syndrome: Results from a national survey of U.S. adults
Source: PLoS One. 2021 Jan 14;16(1):e0245323. doi: 10.1371/journal.pone.0245323 (PMC7808669; doi:10.1371/journal.pone.0245323)
Supplement: S1 File — (DOCX) [file pone.0245323.s001.docx]

**S1 File.**

**Sample**

All participants completed a part I diagnostic interview which utilized the World Health Organization (WHO) Composite International Diagnostic Interview (CIDI) to generate diagnoses based on the DSM-IV. A subset of the participants (n=5,692) also completed a part II interview that included questions about physical conditions (including GI complaints and sleep disturbances), and functional impairments. The part II interview was administered to all participants who met lifetime criteria for any core DSM disorder as well as an approximately one-in-three probability subsample of other participants to increase generalizability. The GI and other physical conditions were inquired in the part II interview which is used in this report. The NCS-R was weighted for non-responders and in addition a part II selection weight was applied. The NCS-R oversampled racial minorities, females, residents of the Midwest, people with ≥13 years of education, and residents of metropolitan areas. These biases were corrected with complex weighting techniques.

**Statistical Analysis**

The Taylor series design-based variance-covariance matrices were used for variance estimates and design-adjusted Wald χ2 tests were used to examine differences across IBS status. For functional impairment analysis, WHODAS-II scores showed skewed distribution. Therefore, we used dichotomized scores for the current analyses; a score ≥95th percentile of the WHODAS-II scores distribution was considered significant impairment. The decision to use the 95th percentile as the cut-off was made in order to capture a proportion of the sample with clinically relevant functional impairment.
